# Exploration of biomedical knowledge for recurrent glioblastoma using natural language processing deep learning models

**DOI:** 10.1186/s12911-022-02003-4

**Published:** 2022-10-13

**Authors:** Bum-Sup Jang, Andrew J. Park, In Ah Kim

**Affiliations:** 1 Department of Radiation Oncology, Seoul National University Hospital, Seoul, Korea; 2 Artificial Intelligence Research and Development Laboratory, SELVAS AI Incorporation, Seoul, Korea; 3 Department of Radiation Oncology, Seoul National University Bundang Hospital, 173 Gumiro, Bundanggu, 13620 Seongnamsi, Kyeonggido Korea; 4 Department of Radiation Oncology, College of Medicine, Seoul National University, Seoul, Korea

**Keywords:** Natural language processing, Recurrent glioblastoma, Name entity recognition, Question and answering, Deep learning, BERT

## Abstract

**Background:**

Efficient exploration of knowledge for the treatment of recurrent glioblastoma (GBM) is critical for both clinicians and researchers. However, due to the large number of clinical trials and published articles, searching for this knowledge is very labor-intensive. In the current study, using natural language processing (NLP), we analyzed medical research corpora related to recurrent glioblastoma to find potential targets and treatments.

**Methods:**

We fine-tuned the ‘SAPBERT’, which was pretrained on biomedical ontologies, to perform question/answering (QA) and name entity recognition (NER) tasks for medical corpora. The model was fine-tuned with the SQUAD2 dataset and multiple NER datasets designed for QA task and NER task, respectively. Corpora were collected by searching the terms “recurrent glioblastoma” and “drug target”, published from 2000 to 2020 in the Web of science (N = 288 articles). Also, clinical trial corpora were collected from ‘clinicaltrial.gov’ using the searching term of ‘recurrent glioblastoma” (N = 587 studies).

**Results:**

For the QA task, the model showed an F1 score of 0.79. For the NER task, the model showed F1 scores of 0.90 and 0.76 for drug and gene name recognition, respectively. When asked what the molecular targets were promising for recurrent glioblastoma, the model answered that RTK inhibitors or LPA-1 antagonists were promising. From collected clinical trials, the model summarized them in the order of bevacizumab, temozolomide, lomustine, and nivolumab. Based on published articles, the model found the many drug-gene pairs with the NER task, and we presented them with a circus plot and related summarization (https://github.com/bigwiz83/NLP_rGBM).

**Conclusion:**

Using NLP deep learning models, we could explore potential targets and treatments based on medical research and clinical trial corpora. The knowledge found by the models may be used for treating recurrent glioblastoma.

## Background

Machine learning or rule-based natural language processing (NLP) models have been used to extract clinical information in clinical oncology. Gupta et al. [1] demonstrated that feature-engineered NLP model achieved promising accuracy in classifying immune-related adverse event from clinical notes of electronic health records. Alkaitis et al. [2] developed the logistic regression and convolutional neural network NLP models to identify treatment discontinuation within a cohort of 6,115 patients with early-stage and 701 patients with metastatic breast cancer. More recently, in the advent of modern deep learning-based NLP models such as the Bidirectional Encoder Representations from Transformers (BERT), application of NLP models in critical or novel medical domains has emerged. For example, Esteva et al. [3] established the coronavirus disease of 2019 (COVID-19) information retrieval system that includes semantic search, question answering, and abstractive summarization. The system was based on Siamese-BERT [4] with the COVID-19 open research dataset.

Glioblastoma (GBM) is one of the lethal tumors, which is mostly managed with a multimodality approach including surgical resection, radiation therapy (RT), and adjuvant chemotherapy [5]. A high rate of recurrence after initial treatment is commonly observed, and progressive or recurrent patients show poor median survival of less than 1 year [6]. Given that there is no standard treatment guideline for patients with recurrent GBM, a consideration of salvage treatment is determined based on multi-disciplinary approach. Though conventional approach with reoperation, reirradiation, or other chemotherapy is possible, additional treatment could increase treatment-related morbidity or mortality in patients with recurrent GBM. Thus, novel approaches and therapeutics are an unmet need. Indeed, patients with recurrent GBM are often recommended to participate in clinical trials of new therapies [7]. Since various novel therapeutics are evolving and being tested, clinicians need to be aware of reasonable approaches based on recent results of translational studies or phase I/II trials. Also, researchers can start their own new study from the results of previous references. Thus, efficient exploration of knowledge for the treatment of recurrent GBM is pivotal for both clinicians and researchers. A vast majority of medical corpora, including the description of all currently activating clinical trials and published articles, can be handled by NLP algorithm.

Recently, modern deep learning-based NLP algorithms have emerged beyond rule-based NLP algorithms [8]. With the advent of transformer models such as the BERT [9], the performance of information extraction from corpora has significantly improved. BERT can perform several tasks, including name entity recognition (NER), question answering (QA), summarization, translation, text classification and text generation [10]. However, BERT is trained based on the general domain corpora. To capture complex semantic contexts in the biomedical domain, the BERT for biomedical domain models such as the BioBERT [11], the BlueBERT [12], or the PubMedBERT [13] have been developed based on the PubMed articles, and they demonstrated better performance in terms of biomedical tasks, compared with BERT for general domain. In particular, the self-aligning pretrained BERT (SAPBERT) [14], which was pre-trained on the biomedical knowledge graph of the unified medical language system (UMLS) [15], outperformed the biomedical domain-specific BERT such as the BioBERT, the BlueBERT, or the PubMedBERT. We hypothesized that the SAPBERT had many advantages in recognition of domain-specific terminology, such as gene, target, drug, and other treatment modalities for recurrent GBM.

Thus, based on the SAPBERT model, we aimed for developing knowledge exploration platform for novel treatment, target, clinical trials in patients with recurrent GBM.

## Methods

We developed two main models for NER and QA tasks by fine-tuning the previously published NLP model based on annotated biomedical corpora. Then, we processed medical corpora relevant to recurrent glioblastoma by developing models and outputs that were summarized and implemented in the user-platform.

### Model selection, data preparation, and fine-tuning

Pretrained models were searched and fine-tuned with the transformer library [16]. We chose the SAPBERT (https://huggingface.co/cambridgeltl/SapBERT-from-PubMedBERT-fulltext) as the base pretrained model. Downloading of the SAPBERT base model, training data preprocess, and fine-tuning was performed by the Transformers library version 4.7.0 and its tutorial notebooks.

To collect research articles, we accessed the Web of Science on March 1, 2021, with the Endnote version 20 programs. In search mode, we used several terms as search conditions as following: “Year: 2000–2020, Title: recurrent glioblastoma AND drug target”. Altogether 288 open access research articles were found, and we retrieved them as a the PDF file format. Then, each PDF document was converted into the JSON file format as structured text by using the publicly available S2ORC software (https://github.com/allenai/s2orc-doc2json). We collected all body text part, composed of altogether 9,950 paragraphs across references. For clinical trials, we found a total of 587 trials related to recurrent GBM from the clinical trial database (https://clinicaltrials.gov) on March 15, 2021. To download detail descriptions of clinical trials, we used the expression term as “recurrent glioblastoma” in the application programming interface mode within the website. Detail information for 587 clinical trials was downloaded in the format of XML (Extensible Markup Language) file. We parsed the XML files and separated the ‘description’ part. For drug and gene NER tasks, we sought data corpora for the drug and gene name. In the public repository (https://github.com/BaderLab/Biomedical-Corpora), we found multiple collections of annotated, freely distributable, biomedical corpora, and CoNLL-like corpora. Data corpora for the gene NER task includes followings: BC2GM_BIO, BioNLP09_BIO, BioNLP11EPI_BIO, BioNLP11ID_BIO, BioNLP13CG_BIO, BioNLP13GE_BIO, BioNLP13PC_BIO, CRAFT_BIO, Ex-PTM_BIO, and JNLPNA_BIO. Meanwhile, data corpora for the drug NER task includes followings: BC4CHEMD_BIO, BC5CDR_BIO, BioNLP11ID_BIO, BioNLP13CG_BIO, and BioNLP13PC_BIO. Finally, we collected 32,258 and 41,043 entities for drug and gene name, respectively. Of those, training set was defined as the sum of the training and validation entities, and the testing set was defined as the original testing entities.

To fine-tune NER task, two NER task models fitted for gene and drug name were developed by following fine-tuning parameters: Learning rate = 5e-5, batch size = 16, number of running epochs = 5, warm-up step = 500, weight decay = 0.1. To fine-tune QA task, we achieved the whole SQUAD2 dataset and divided it into training (N = 130,319) and testing sets (N = 11,873). Fine-tuning parameters included following: Batch size = 16, max length = 512, learning rate = 2e-5, number of running epochs = 4, weight decay = 0.01.

### Implementation of knowledge platforms using trained models

To implement the platform from QA task models, we adopted a document retriever and summarizer. We used the Elasticsearch version 7.1.3 (https://www.elastic.co/elasticsearch) as a document retriever that indexes and searches appropriate body texts by queries from research articles. The BM25 is the default similarity ranking algorithm according to relevancy with queries in the Elasticsearch. Thus, we sorted selected body text according to the BM25 and used them as input for summarization model. We adopted the BART large CNN model (https://huggingface.co/facebook/bart-large-cnn) as the document summarizer. To summarize the collected answers and make the final answer, we used the following parameters for the BART model: the number of beams = 5, length penalty = 1.2, max length = 256, minimum length = 128, and the number of repeat ingram sizes = 5.

To implement the platform from NER task models, we filtered and counted single sentence that contained both drug and gene name from research articles. Generated drug-gene pairs were presented as a circus plot. For clinical trial text, we extracted drug names and sorted them. Given that one drug corresponded to multiple clinical trials, a summary of their descriptions was also provided by the BART model. All visualization and user-interface were organized using Microsoft Power BI Desktop version 2.96 (http://app.powerbi.com).

## Results

### Fine-tuned model performance

The performance of fine-tuned model is summarized in Table 1. Overall, the drug NER model showed better performance than the gene NER model. In terms of accuracy, both the drug and gene NER model demonstrated similar performance (0.993 vs. 0.968). However, there was a difference in precision between the two models (0.912 for drug NER vs. 0.715 for gene NER). This difference was linked to the difference in F1-score: 0.908 and 0.760 for drug and gene NER model, respectively. On SQUAD2 test dataset, fine-tuned model for the QA task showed an F1-score of 0.792 and an exact match of 0.758.

**Table 1:** Evaluation Results of Fine-tuned Models

	Drug NER	Gene NER	QA task
**Precision**	0.912	0.715	N/A
**Recall**	0.904	0.811	N/A
**Accuracy**	0.993	0.968	N/A
**F1**	0.908	0.760	0.792
**Exact Match**	N/A	N/A	0.758

### Implementation of model

**Fig. 1:**
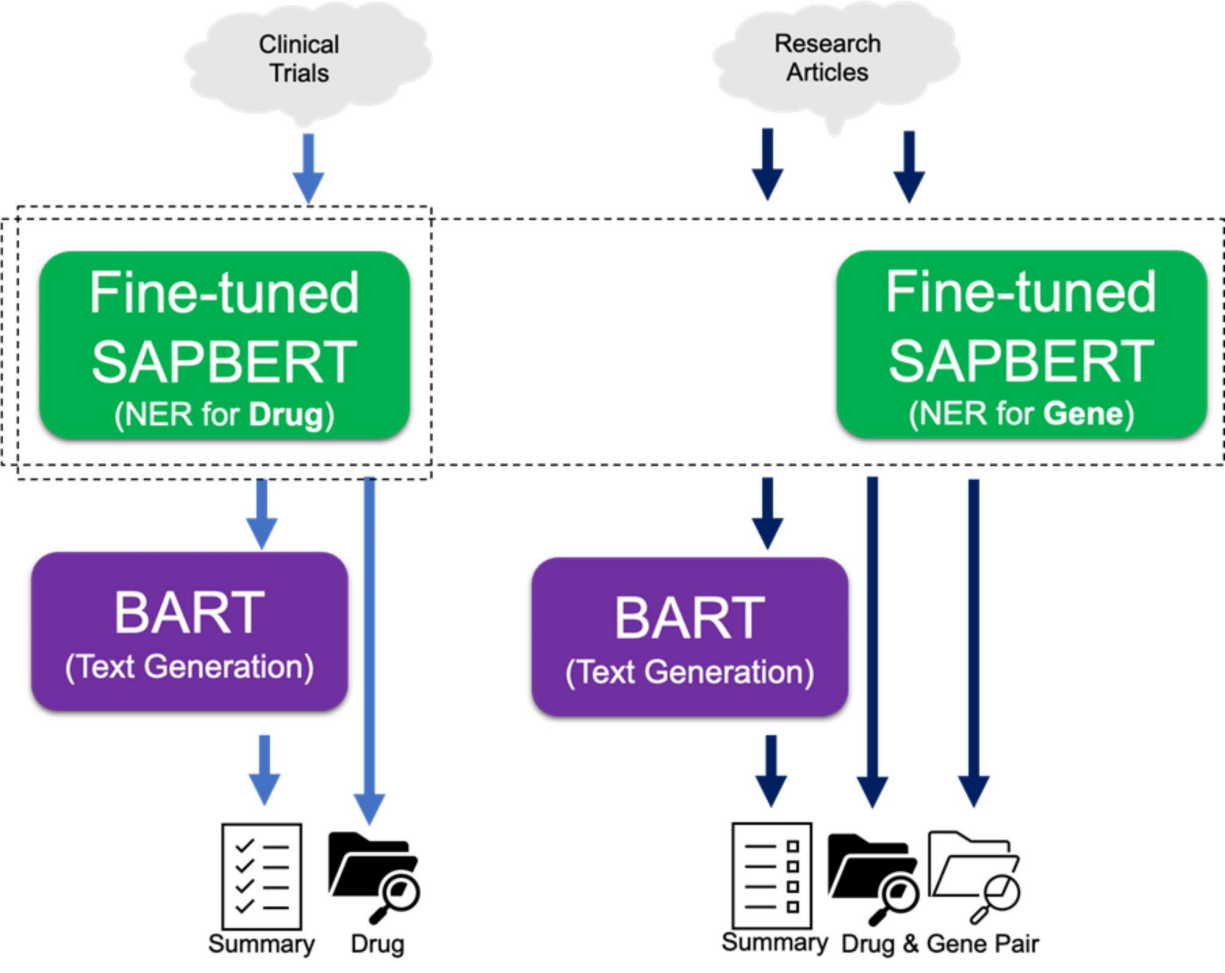
The overflow of NER-based knowledge exploration platform. NER, name entity recognition; SAPBERT, Self-aligning pretrained BERT.

An overview of the NER task is depicted in Fig. 1. Drug NER model extracted the names of drugs from the descriptions of clinical trials. The relationship between the drug and the clinical trial was a one-to-many relationship. Then, we could summarize multiple descriptions with the BART model. From research articles, both drug and gene NER models were used to extract drug-gene pair. Since the relationship between drug-gene pair and research articles was a one-to-many relationship, we could provide an abstract summary from multiple articles that include a certain drug-gene pair by using the BART model.

**Fig. 2:**
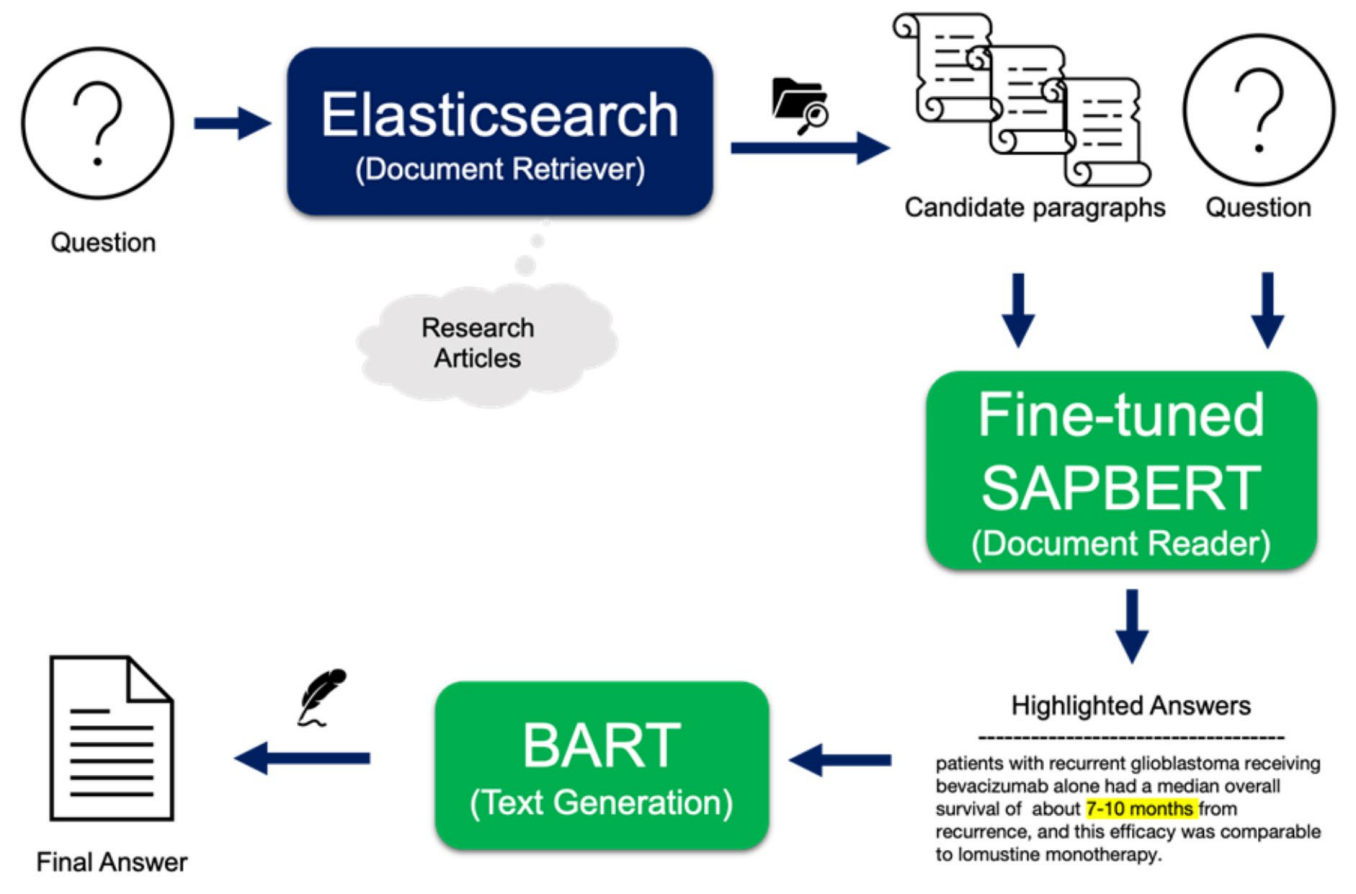
The overflow of QA-based knowledge exploration platform. QA, question and answer; NER, name entity recognition; SAPBERT, Self-aligning pretrained BERT.

The flow of the QA task is visualized in Fig. 2. According to the question, document retriever selects multiple potential answer paragraphs from indexed research article database. Then, fine-tuned QA task model finds a precise answer phrase in each graph. Multiple sentences containing an answer phrase are summarized by the BART model to obtain the final answer. Based on this workflow, example questions that are clinically challengeable issues in recurrent GBM and their corresponding answers are listed in Table 2.

**Table 2:** Example questions and their answers generated by models

Query	Generated and Summarized Answer by Models
What is a reirradiation dose/fractions for recurrent glioblastoma?	The most common approach involves the use of fractionated stereotactic radiotherapy with or without intensity modulation and a median total dose of 30–36 gy. Stereotactic radiosurgery (the administration of one single fraction), is rarely used in glioblastoma because of the poorly defined target volume. None of the reirradiation schedules has ever been looked at in a prospective or controlled fashion. The recent apg101 trial provided no sign of efficacy for reir Radiation at × 2 gy in recurrent glioma patients. temsirolimus administered weekly at the dose of mg / m 2 also did not show efficacy in children with high-grade gliomas.
What is median overall survival in patients with recurrent glioblastoma?	Bevacizumab alone had a median overall survival of about 7–10 months from recurrence, and this efficacy was comparable to lomustine monotherapy. For patients with recurrent glioblastoma, chemotherapy regimens are associated with overall response rates of 4–9%, 6-month progression-free survival (pfs) of 10–19%. Even with these treatments, median overall survival after recurrence is 6. 2 months. fda-approved treatment options remain few and the prognosis remains dismal with a median survival of 14. 6 months and a 5-year-survival rate of 9.
Are immune checkpoint inhibtors are available for patients with recurrent glioblastoma?	There are many ongoing clinical trials with immune checkpoint inhibitors in patients with primary and recurrent glioma / glioblastoma. challenges in the design and conduct of clinical trials for immunotherapies are numerous, particularly in trials involving patients with gliOBlastomas. different measures of response are required for checkpoint inhibitors, and the management of immune-related adverse events in the cns are a concern. as immunotherapy becomes more widely available, the potential increases for both synergies and adverse interactions between conventional gliobeastsoma therapies and immune checkpoint inhibitor. There are currently limited data on immune checkpoints in other types of gliomas such as oligodendroglioma.
What molecular targets are potentially promising for recurrent glioblastoma?	Molecular therapies that targeted rtks are promising therapeutic strategies for glioblastoma tumors. Clinical trials have not shown promising combinational therapies of temsirolimus with bevacizumab (vegf inhibitor), sorafenib (raf inhibitor), erlotinib (egfr inhibitor), or radiation therapy. The molecular target expression status, as determined at the time of primary resection, may not necessarily present rational treatment clues for the care of recurrent gbm that occurs 6–9 months later. The lpa 1 antagonist ki16425 (kirin brewery co., takasaki, japan) effectively suppresses the lpa-induced motility of gliobeasts.
Is MGMT status associated with the incidence of recurrent glioblastoma?	Methylated mgmt status determined by msp was correlated with better outcome. The prognostic value of the mgMT status in patients with recurrent glioblastoma is not well defined. Future research will shed light on which patients should undergo a second resection or radiotherapy procedure. It will also shed light how to best use tmz and bevacizumab therapy, and the value of mgmt Status assessment in the recurrent setting. The study also found that mgmtstatus did not appear to change between primary and recurrent tumors. It is positively associated with gliOBlastoma sensitivity to alkylating agents, such as temozolomide.
What is the pattern of care in recurrent glioblastoma?	Radiotherapy remains an important part of the standard-of-care treatment for patients with malignant gliomas. Despite definitive data, standard of care guidance for managing patients with recurrent or progressive glioblastoma is evolving. The diffusely infiltrative pattern of progression might be associated with a slower cause of the disease, as it has been suggested by radiological patterns of recurrence of patients treated with bevacizumab. The 6-month pfs rate (pfs6) is the optimal end point for treatment of recurrent gliOBlastoma. Participation in clinical trials is encouraged for the treatment of this type of cancer. The primary purpose of this paper is to discuss the role of second-line monotherapy and combination therapies.

**Fig. 3:**
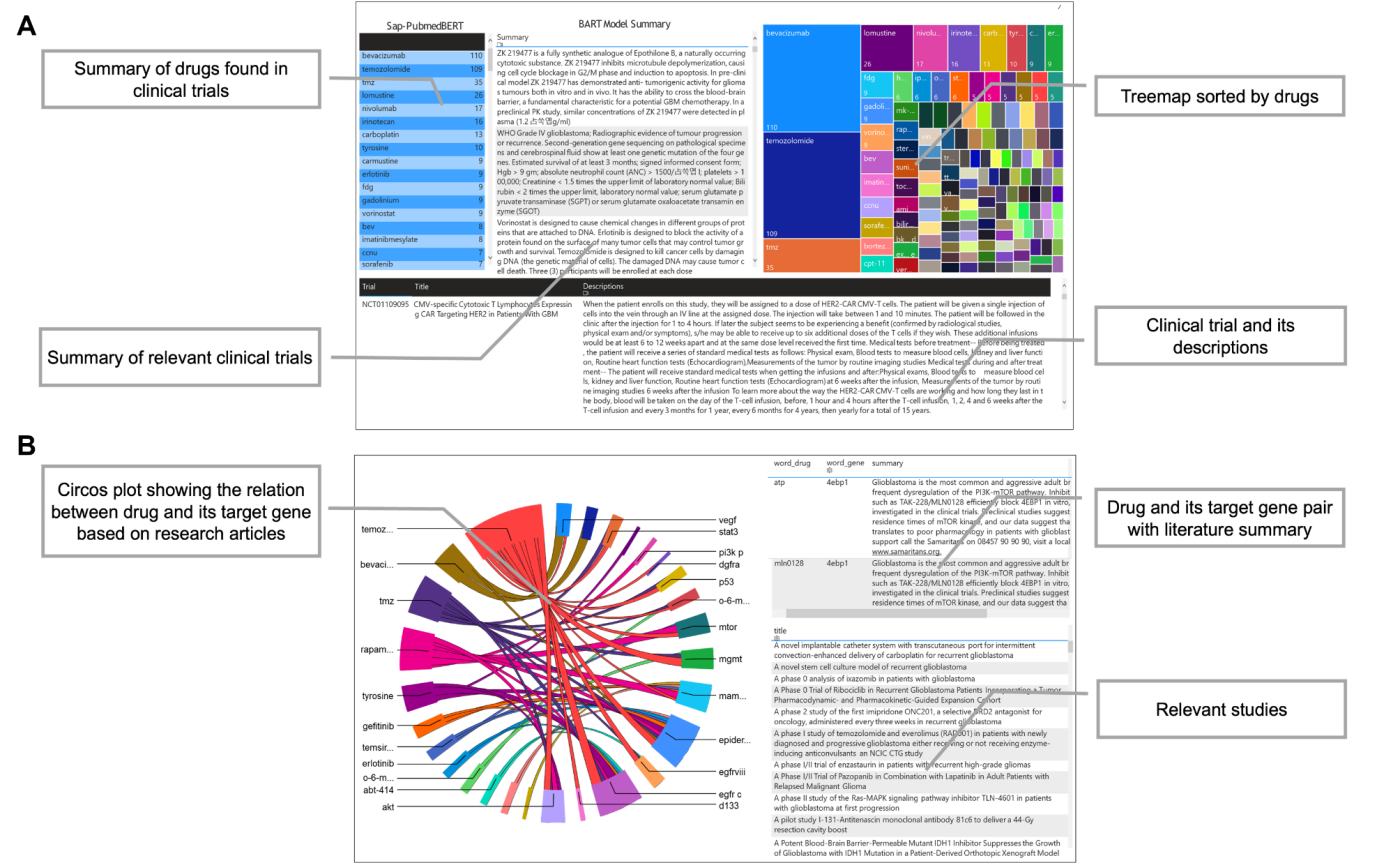
Visualization of results from NER task by models. **(A)** Extracted drug names from clinical trials and their presentation. Drug names are displayed in the tree map in order of frequency. **(B)** Extracted drug-gene pairs from research articles are presented in circus plot. For each drug-gene pair, abstracts of relevant articles are summarized

NER model implementation platforms for clinical trials and research articles are visualized in Fig. 3. For clinical trial exploration, fine-tuned model extracted drug names from the descriptions of clinical trials. Then, we matched drug and relevant trials, and demonstrated their link in real-time manner. End-users can easily find the most cited drugs within clinical trials by accessing a tree map. Descriptions of multiple trials matched with each drug are summarized by the BART model, and their summaries are provided to users (Fig. 3 A). For research article exploration, two fine-tuned models were employed to extract both the drug and gene name in parsed paragraphs. We generated drug-gene pair when they exist in one sentence. Then, drug-gene pairs are presented as a circus plot (Fig. 3B). Relevant articles and their abstract summaries are displayed in real-time manner when clicking on a drug, which was mentioned more than 2 times among all drug-gene pairs.

## Discussions

We constructed the platforms that can discover a recent medical knowledge about recurrent GBM. After fine-tuning biomedical-specific BERT models for NER tasks, we extracted drug-gene pairs from research articles and clinical trials and could construct NER-based knowledge platform. Following fine-tuning QA models, we also established QA-based knowledge platform. This work may help researchers easily find novel targets and clinicians make decisions or matching clinical trials for recurrent GBM patients. This study is the first to use fine-tuned NLP models for oncologists in the treatment of recurrent GBM.

In the current study, we fine-tuned the SABPERT [14] that was expected to suitable for information retrieval of potential biomarkers, treatment, and any relevant knowledge elicited by QA-based task for recurrent GBM. According to evaluation metrics, the model performance seems to follow the pretrained model performance well in terms of the NER task. However, F1 score was only 0.79 in the QA task, and there are several reasons to explain the relatively low performance. The SAPBERT was not validated in the QA task but demonstrated high accuracy in the NER task. Also, fine-tuning dataset for the QA task was the SQuAD dataset which was consisted of questions based on a set of Wikipedia articles. We speculate that the model performance for medical QA task might be improved when a biomedical-specific QA dataset such as BioASQ was used for fine-tuning. However, this process will accompany dataset conversion process, which would be labor-intensive. Although we could not demonstrate the data, we compared and benchmarked other BERT-based models including the BioBERT and the BERT. The difference was not that significant, however, the SAPBERT demonstrated the slight superior performance.

For patients with recurrent GBM, clinical trials are the preferred options. Otherwise, the reuse of current therapies is individualized according to the performance status, quality of life, and overall prognosis [7]. Based on the expected median survival and relevant prognostic factors from each patient, radiation oncologists individualize prescription dose and fractions as long as reirradiation is possible. However, the prognostic and predictive factors for patients recurrent GBM is still obscured. From the clinical perspective of oncologists, these clinical issues may be elucidated with cumulating domain knowledge. Regarding several clinical challenges for recurrent GBM, we could summarize the generated answers from the QA-based knowledge platform as follows: Although radiotherapy is the standard treatment for primary GBM, there is no standard care for recurrent GBM. When reirradiation is suggested, median dose of 30-36 Gy with fractionation is the common approach for recurrent GBM patients. The median overall survival is 7–9 months with bevacizumab alone, and there are few in the U.S. The Food and Drug Administration approves drugs for recurrent GBM. The generated answer indicated that receptor tyrosine kinase (RTK) or Lysophosphatidic acid receptor-1 (LPA-1) is potential target for treating recurrent GBM. Indeed, re-irradiation for the recurrent glioblastoma was limited to a dose of 24 to 36 Gy with a daily fractional size of 1.8 to 6 Gy [17]. In a randomized trial comparing the regimen of systemic therapies in recurrent glioma [6], median overall survival was 9.1 months and 8.6 months in the addition of bevacizumab to the lomustine group and the bevacizumab alone group, respectively. Also, cumulating literature [18–20] addressed that LPA-1 antagonist could be a promising approach since LPA-1 expression is high in GBM and promotes GBM proliferation and migration.

Information about new agent such as immunotherapy and novel targets is important for oncologists when encouraging the clinical trials for patients. Drugs that appeared frequently in clinical trials were bevacizumab, temozolomide, followed by lomustine. When focusing on immunotherapy, models revealed that nivolumab, anti-PD-1 inhibitor, is the most mentioned drug in current clinical trials for recurrent GBM. However, immunotherapy is not recommended routinely, based on the results of several immunotherapy trials such as the CheckMate 143 [21]. Nevertheless, we should be noted that there are ongoing efforts to reveal patient subgroups that could show a good response to other immunotherapy agents with a combination with radiation [22]. Furthermore, the model revealed that, in clinical trials, temozolomide targeted relevant genes including Akt, CD133, the epidermal growth factor receptor (EGFR), EGFR variant III (EGFRvIII), o6-methylguanine-dna methyltransferase (MGMT), and mammalian target of rapamycin (mTOR). Meanwhile, the most targeted gene was EGFR, which was mentioned altogether 79 times in the platform. Indeed,  the INTELLANCE 2/EORTC 1410 randomized phase II trial [20] showed that EGFR monoclonal antibody conjugated to a tubulin inhibitor and temozolomide showed an improved survival compared to lomustine or temozolomide alone (median overall survival 9.6 month vs. 8.2 month), albeit which was not statistically significant.

There are several limitations in the current study. We could not address that fine-tuned NLP models understood the meaning of medical terminology as like expert oncologists. Thus, generated answers and summaries shroud be cautiously interpreted, and relevant references should be verified. In terms of clinical trials, note that this study aimed to provide a brief of current or potential eligible trials efficiently. A review of many factors, such as patient eligibility criteria, should be considered by appropriate experts. The NLP modes used in the current study coud not discern abbreviations such as EGFR or epidermal growth factor receptor. To resolve this issue, hand-crafted or rule-based trimming may be needed. For fine-tuning task, the general domain QA dataset was used instead of biomedical-specific QA dataset such as BioASQ. Fine-tuning with a more specific type of dataset may show more improved performance.

## Conclusion

In conclusion, we established platforms for oncologists or researchers based on fine-tuned deep learning-based NLP models to discover medical knowledge from recently published articles and ongoing clinical trials for recurrent GBM. This could help decision-making process regarding the consideration of further treatment or encouraging clinical trials for patients with recurrent GBM.

## Data Availability

The datasets used and/or analysed during the current study available from the corresponding author on reasonable request. However, source codes and processed results from NLP models can be found at https://github.com/bigwiz83/NLP_rGBM.

## List of abbreviations

NLP: natural language processing.
NER: Aname entity recognition.
QA: question answering.
GBM: glioblastoma.
RT: radiation therapy.
BERT: Bidirectional Encoder Representations from Transformers.
SAPBERT: self-aligning pretrained BERT.
UMLS: unified medical language system.
RTK: receptor tyrosine kinase.
LPA-1: lysophosphatidic acid receptor-1.
EGFR: epidermal growth factor receptor.
MGMT: O6-Methylguanine-DNA Methyltransferase.
